# Supplementary material for: Patterns of Intron Gain and Loss in Fungi
Source: PLoS Biol. 2004 Nov 30;2(12):e422. doi: 10.1371/journal.pbio.0020422 (PMC532390; doi:10.1371/journal.pbio.0020422)
Supplement: Table S1 — Also available at http://genes.mit.edu/NielsenEtAl/. (4.3 MB ZIP). [file pbio.0020422.st001.zip › NielsenEtAl/html/765.html]

AN0164.1.NCU06563.1.MG01690.1.FG05281.1


```
 CLUSTAL W (1.82) Multiple Sequence Alignments - Introns Inserted


Sequence 1: MG01690.1	299 aa
Sequence 2: FG05281.1	299 aa
Sequence 3: NCU06563.1	281 aa
Sequence 4: AN0164.1	299 aa
Alignment Length: 299 aa
Number Identitical Residues: 238 aa
Alignment Score (without introns) 10334


MG01690.1 	MLAESVIEAICAKTKELLMRESNVVHVRAPVTVVGDIHGQFFDLIEIFKIGGWCPDTNYL
NCU06563.1	------------------MRESNVVHVRAPVTVVGDIHGQFYDLIEIFRIGGYCPDTNYL
FG05281.1 	MLAESVIEAVCAKTKELLMRESNVVHVRAPITVVGDIHGQFFDLIEIFRIGGYCPDTNYL
AN0164.1  	MLADSVIEAICAKAKELLMKESNVVHIAAPVTVVGDIHGQFFDMIEIFKIGGFCPNTNYL
          	  :.:  .: .:.:..  *:******: **:**********:*:****:***:**:****

MG01690.1 	FLG1DYVDRGMFSVETISLLVCLKLRYPNRVHLIRGNHESRGVTQSYGFYTECSRKYGNA
NCU06563.1	FLG1DYVDRGMFSVETISLLVCLKLRYPNRVHLIRGNHESRGVTQSYGFYTECSRKYGNA
FG05281.1 	FLG1DYVDRGMFSVETISLLVCLKLRYPNRVHLIRGNHESRGVTQSYGFYTECSRKYGNA
AN0164.1  	FLG1DYVDRGLFSVETISLLVCLKLRYPSRVHLIRGNHESRGVTQSYGFYTECARKYGNA
          	*** ******:*****************.************************:******

MG01690.1 	NVWHHFTDMFDFLTLSVVINDQIFCVHGG1LSPSIHSIDQIKIIDRFREIPHEGPMADLV
NCU06563.1	NVWHYFTDMFDFLTLSVVINDQIFCVHGG1LSPSIHSIDQIKVIDRFREIPHEGPMADLV
FG05281.1 	NVWHYFTDMFDFLTLSVVINDQIFCVHGG1LSPSIHSIDQIKIIDRFREIPHEGPMADLV
AN0164.1  	NVWHYFTDMFDFLTLAVVINDQIFCVHGG~LSPSIHSIDQIKIIDRFREIPHEGPMADLV
          	****:**********:************* ************:*****************

MG01690.1 	WSDPDPERDEFSLSPRGAGYTFGAQVVKKFLAVNGMSHILRAHQLCQEGYQVLYDDRLST
NCU06563.1	WSDPDPDRDEFSLSPRGAGYTFGAQVVKKFLAVNNMNHILRAHQLCQEGYQVLYDDRLST
FG05281.1 	WSDPDPERDEFSLSPRGAGYTFGAQVVKKFLAVNNMSHILRAHQLCQEGFQVLYDDRLST
AN0164.1  	WSDPDTERDEFSLSPRGAGYTFGAQVVRKFLEVNSMSHILRAHQLCQEGYQVLYDDRLST
          	*****.:********************:*** **.*.************:**********

MG01690.1 	VWSAPNYCYRCGNMASVLEVSDTGERFFNVFAAAPENDQHKDIQPGGEKSADSSALPDYF
NCU06563.1	VWSAPNYCYRCGNMASVLEVSDTGERFFNVFAAAPENDAVKDQQMNQDKSAEGGALPDYF
FG05281.1 	VWSAPNYCYRCGNMASVLEVSDTGERFFNVFAAAPENDVHKDLQPGNEKSADGNALPDYF
AN0164.1  	VWSAPNYCYRCGNLASVLEVSDTGERFFNIFDAAPENDIHRGEQQTQQSKDGQGPVIDYF
          	*************:***************:* ******  :. *   :..   ..: ***

MG01690.1 	L
NCU06563.1	L
FG05281.1 	L
AN0164.1  	L
          	*
```
